# A Coupling Kinetics Model for Pollutant Release and Transport in the Process of Landfill Settlement

**DOI:** 10.3390/ijerph9103437

**Published:** 2012-09-27

**Authors:** Ying Zhao, Qiang Xue, Lei Liu

**Affiliations:** State Key Laboratory of Geomechanics and Geotechnical Engineering, Institute of Rock and Soil Mechanics, Chinese Academy of Sciences, Wuhan, Hubei 430071, China; Email: yzhao@whrsm.ac.cn (Y.Z.); lgdliulei@163.com (L.L.)

**Keywords:** municipal solid waste, landfill leachate pollutants, aerobic and anaerobic degradation, dissolved oxygen, settlement, coupling kinetics model

## Abstract

A coupling kinetics model is developed to simulate the release and transport of landfill leachate pollutants in a deformable municipal solid waste landfill by taking into account of landfill settlement, seepage of leachate water, hydrolyse of insoluble and degradable organic pollutants in solid phase, biodegradation of soluble and degradable organic pollutants in solid phase and aqueous one, growth of aerobic and anaerobic microorganism, and consumption of dissolved oxygen. The release and transport of organic pollutants and microorganisms in landfills in the process of landfill settlement was simulated by considering no hydraulic effect. Simulation results demonstrated that the interaction between landfill settlement and the release, transport and biodegradation of landfill leachate pollutants was significant. Porosity and saturated hydraulic conductivity were not constants because of the landfill settlement, which affected the release, transport and biodegradation of landfill leachate pollutants, and furthermore acted on the landfill settlement. The simulation results accorded with the practical situation, which preliminarily verified the reliability of the mathematical model and the numerical program in this paper.

## 1. Introduction

The release and transport of landfill leachate is a complex process, affected by landfill settlement, fluid movement, biodegradation and temperature changes, so a complete model which describes the release and transport of landfill leachate pollutants must contain mechanical, hydraulic, gas transport, temperature and biodegradation models, but it is almost impossible to realize a five-field coupling simulation, so this is often simplified to two or three field coupling model.

Many researchers have studied the multi-field coupling problems of landfill leachate transport, and new models have been developed based on more detailed mathematical descriptions of the landfill and incorporating other aspects of interest apart from hydrology, such as the biological and physical-chemical degradation and settlement. Demirekler *et al.* [1] developed a three-dimensional mathematical model to estimate the quality and quantity of the landfill leachate produced. The effect of overburden stress was considered. Lobo *et al. *[2,3] has reported the first version of MODUELO. The development of the second version and the Meruelo Landfill (Spain) simulation results were presented in Lobo *et al.* [4,5,6]. Chanthikul *et al.* [7] developed a mathematical model of BOD5 concentration without and with leachate recirculation. Durmusoglu *et al.* [8] developed a one-dimensional multiphase numerical model to simulate the vertical settlement involving liquid and gas flows in a deformable MSW landfill. McDougall [9] developed a hydro-bio-mechanical model for settlement and other behavior in land filled waste. Fellner and Brunner [10] established a 2-dimensional 2-domain model for simulating the leachate generation from MSW landfills. A flow field consisting of a vertical path (channel domain) surrounded by the waste mass is defined using the software HYDRUS-2D. One-dimensional advection–dispersion transport modeling was conducted as a conceptual approach for the estimation of the transport parameters of fourteen different phenolic compounds and three different inorganic contaminants migrating downward through the several liner systems in Gamze *et al.* [11]. Gaetano *et al.* [12] presents a 1D mathematical model for the simulation of the percolation fluxes throughout a landfill for MSW, which considered the landfill divided in several layers evaluating the inflow to and outflow from each layer as well as the continuous moisture distribution. But, there are still one or more defects in most models as follows: (a) The pollutants were considered as a single solute, whether it is soluble or insoluble, degradable or non-degradable was not definitely differentiated; (b) Solid-phase pollutants and its dissolution to aqueous phase weren’t taken into account; (c) The effect of oxygen on biodegradation was neglected and the transition from aerobic degradation to anaerobic one wasn’t taken into account; (d) the effect of settlement on porosity, saturated hydraulic conductivity and the transport of pollutant was neglected. 

A coupling kinetic model was developed to simulate the release and transport of leachate pollutants in a deformable MSW landfill taking into account of hydrolyse and dissolution of solid-phase pollutants, oxygen consumption and transition of aqueous-phase pollutant biodegradation from anaerobic stage to aerobic one, and other behaviors such as convection and hydrodynamic dispersion, adsorption/desorption and growth of microorganism. A case study was given by considering none hydraulic action for studying the change law of water quality and quantity, which preliminarily verified the reliability of the mathematical model by comprising with the practical situation.

## 2. Mathematical Model

### 2.1. Basic Assumptions

The release and transport of organic pollutants in landfill is a complicated process which is accompanied by physical behavior and chemical and microbial reactions. It can be barely described by a completely correct model. The development of the simulation model must be based on some suitable assumptions. The assumptions of the models in this study are as follows: (a) Landfill gas is released rapidly after generation, so the landfill leachate transport is considered as a single phase flow; (b) MSW particles are incompressible, but degradable; (c) The simulated landfill was taken as a biochemical reactor. Organics transport and transform under a series of physical, chemical and biological actions, such as convection and hydrodynamic dispersion, hydrolyse, dissolution, adsorption/desorption and biodegradation; (d) Density and viscosity coefficient of landfill lecheate are constants.

### 2.2. Landfill Settlement Model

#### 2.2.1. Mass-Conservation Equation

Based on the mass conservation principle the mass-conservation equation of solid phase is:


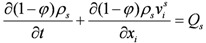
(1)

where 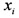 is the space coordinates [L]; 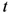 is the current time [T]; 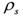 is the solid phase density [ML^−^^3^];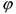 is the porosity; 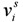 is the velocity of solid phase [LT^−^^1^]; 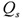 is the source/sink term, which is caused by the degradation of solid waste, the release of inner source water *etc.* [ML^−^^3^T^−^^1^].

#### 2.2.2. Mechanical Model

The Merchant model was used to simulate landfill settlement. It was constructed by a Hooke elastomer and a Kelvin model in series. Kelvin model was constructed by a Hooke elastomer and a Newton viscosity mode in parallel. The creep equation is:


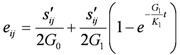
(2)

where 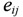 is the deviator strain tensor [LL^−^^1^]; 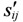 is the deviator stress tensor [ML^−^^1^T^−^^2^]; 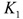 is the viscosity coefficient [ML^−^^1^T^−^^2^]; 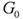 and 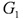 are the shear modulus for Hooke elastomer and Kelvin model, respectively [ML^−^^1^T^−^^2^].

In addition:


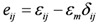
(3)


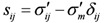
(4)

So the effective stress can be written as:


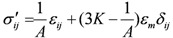
(5)

In Equations (3)–(5), 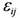 is the strain tensor [LL^−^^1^]; 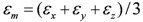 is the mean strain [LL^−^^1^]; 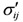 is the effective stress tensor [ML^−^^1^T^−^^2^]; 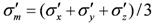 is the mean effective stress [ML^−^^1^T^−^^2^]; 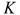 is the bulk modulus [ML^−^^1^T^−^^2^]; 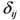 is the Kronecher symbol; and:


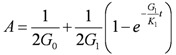
(6)

Furthermore, the effective stress principle can be described by:


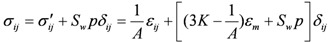
(7)

where 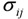 is the total stress [ML^−^^1^T^−^^2^]; 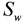 is the liquid saturation [LL^−^^3^]; 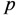 is the liquid pressure [ML^−^^1^T^−^^2^].

Geometric equation and stress equilibrium equation are:


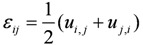
(8)


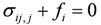
(9)

The stress equilibrium equation represented with displacement can be obtained by plugging Equation (7) and Equation (8) to Equation (9):


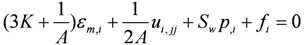
(10)

The velocity of solid phase is:


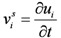
(11)

Equation (1) and Equation (7) (or Equation (10)), Equation (8) and Equation (11) are the basic equations of landfill settlement model. The liquid pressure 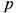 was contained in it, so the hydraulic model must be developed for obtaining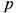.

### 2.3. Hydraulic Model

Based on the mass conservation principle the continuity equation of aqueous phase is:


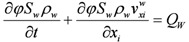
(12)

where 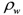 is the liquid phase density [ML^−^^3^]; 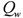 is the source/sink term [ML^−^^3^T^−^^1^]; 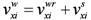 is the absolute velocity of aqueous phase [13] [LT^−^^1^]; and 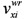 is the relative velocity of aqueous phase to the solid phase [LT^−^^1^].

During settlement, the solid particles as well as the liquid move simultaneously. Hence, it is necessary to state Darcy’s law relative to solids movement. That is:


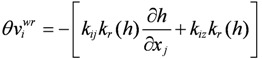
(13)

where 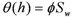 is the volumetric moisture content [L^3^L^−^^3^]; 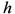 is the hydraulic pressure head [L]; 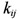 is the saturated hydraulic conductivity tensor [LT^−^^1^]; 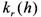 is the relative permeability. 

VG function [14] is used to describe the water retention curve:


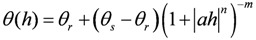
(14)

where 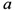, 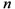, 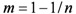 are parameters; 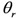 and 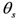 are the residual and saturated volumetric moisture contents, respectively. 

So the relative permeability can be written as:


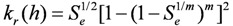
(15)

where 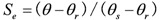 is the effective saturation. 


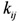
 and 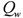 [15] are calculated by: 


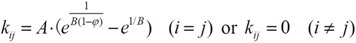
(16)


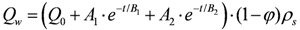
(17)

where 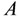, 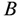, 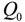, 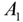, 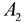, 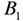 and 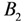 are parameters; 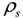 is the particle density of MSW [ML^−^^3^].

### 2.4. Pollutant Release and Transport Model

#### 2.4.1. Conceptual Framework

Organic biodegradation in landfills can be divided into two stages: (1) aerobic biodegradation and (2) anaerobic biodegradation. The first one always occurs in the initial landfill stage, and it can be also divided into two stages: (1) the hydrolysis stage of insoluble macromolecular organics to soluble and small molecular ones and (2) the biodegradation of soluble organics to H_2_O and CO_2_, *etc*. When the oxygen is consumed, biodegradation enters the anaerobic stage. In this stage, macromolecular organics are hydrolyzed to small molecular ones, and then decomposed to CH_4_ and H_2_O by anaerobic microorganisms after the acidification process.

**Figure 1:**
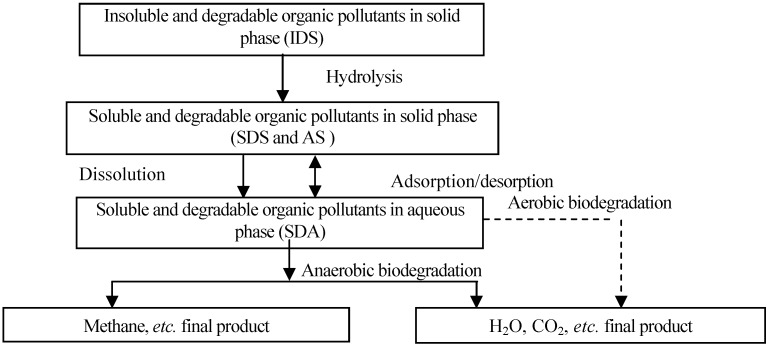
The biodegradation process of organics in landfills.

Based on above biodegradation process, organic pollutants in landfill can be classified as insoluble and degradable ones (IDS), soluble and degradable ones (SDS), and adsorbed ones (AS) in solid phase; and soluble and degradable ones in aqueous phase (SDA). Microorganism includes aerobic and anaerobic ones in aqueous phase (AM and ANM) and hydrolysis ones in solid phase (MS). The model which describes the pollutant release and transport in landfill can be developed by using the mass conservation principle, including hydrolysis of IDS, dissolution and biodegradation of SDS, adsorption/desorption and aerobic and anaerobic biodegradation of SDA; growth and death of AM, ANM and MS, and consumption of dissolved oxygen (DO). The biodegradation process of organics was shown in Figure 1.

#### 2.4.2. Hydrolysis of IDS

The hydrolysis of insoluble macromolecular organics (IDS) to soluble and small molecular ones can be described as first order reaction:


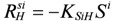
(18)

where 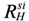 is the hydrolysis rate of IDS [MM^−^^1^T^−^^1^]; 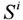 is the concentration of IDS [MM^−^^1^] and 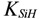 is the hydrolysis constant [T^−^^1^].

#### 2.4.3. Dissolution of SDS

The dissolution of SDS is closely related to the water content and the pollutant concentrations in solid and aqueous phase. It is described by [16]:


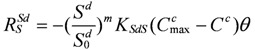
(19)

where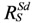 is the dissolution rate of SDS [MM^−1^T^−1^]; 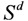 and 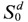 are the concentrations of SDS at time t and initial time, respectively [MM^−1^]; 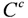 is the concentration of SDA [ML^−3^]; 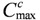 is the maximum concentration of SDA [ML^−3^];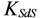 is the dissolution rate constant [ML^−3^T^−1^]; 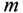 is the dissolution coefficient.

#### 2.4.4. Biodegradation

The decomposition and stabilization of MSW in landfill is essentially a microbial metabolic process. The depletion of the substrate and microorganism growth can be described by Monod kinetics [17], hence for MS accumulation:


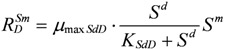
(20)

where 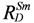 is the growth rate of MS [MM^−^^1^T^−^^1^]; 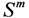 is the concentration of MS [MM^−^^1^]; 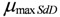 is the maximum specific growth rates for MS [T^−^^1^]; 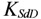 is the half saturation constant for SDS [MM^−^^1^].

The depletion rate of the substrate is directly related to MS accumulation through a cell/substrate yield coefficient 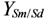:


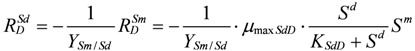
(21)

where 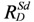 is the depletion rate of SDS [MM^−^^1^T^−^^1^]; the 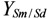 is the stoichiometric yield coefficient for MS (biomass produced per unit amount of electron donor utilized) [MM^−^^1^].

When the dissolved oxygen (DO) exists, and its concentration is low, the cell growth rate for AM and ANM can be represented by the following double Monod models [18]:


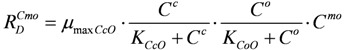
(22)


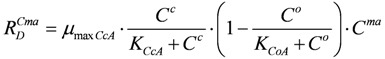
(23)

where 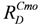 and 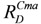 are the cell growth rates for AM and ANM, respectively [ML^−^^3^T^−^^1^]; 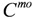 and 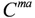 are the concentrations of aerobic microorganism and anaerobic microorganism, respectively [ML^—^^3^]; 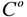 is the DO concentration [ML^−^^3^];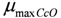 and 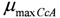 are the maximum specific growth rates for aerobic and anaerobic microorganism, respectively [T^–^^1^]; 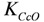 and 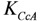 are the half saturation constant for aerobic and anaerobic microorganism, respectively [ML^—^^3^]; 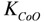 and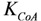 are the half saturation constants for DO [ML^−^^3^].

The depletion rates of the substrate and DO in aqueous phase are directly related to AM and ANM accumulation through cell/substrate yield coefficients 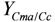, 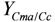 and 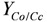:


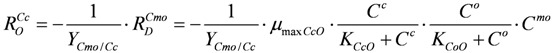
(24)


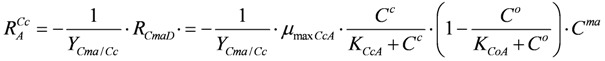
(25)


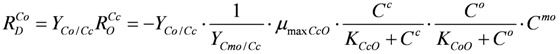
(26)

where 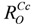 and 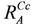 are the aerobic and anaerobic degradation rates of SDA, respectively [ML^−^^3^T^−^^1^]; 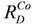 is the consumption rate for DO [ML^−^^3^T^−^^1^]; 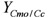 and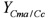 are the stoichiometric yield coefficients for aerobic microorganism and anaerobic microorganism, respectively (biomass produced per unit amount of electron donor utilized); 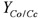 is the consumption coefficient of DO (oxygen consumed per unit amount of SDA). 

The MS, AM and ANM decay are given by:


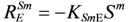
(27)


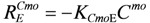
(28)


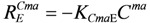
(29)

where 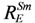, 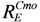 and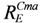 are the endogenous cell death or decay rates of MS, AM and ANM, respectively [ML^−^^3^T^−^^1^]; 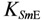, 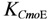 and 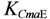 are the endogenous cell death or decay coefficients of MS, AM and ANM, respectively [T^−^^1^].

#### 2.4.5. Adsorption/Desorption of SDA

Langmuir adsorption model can describe the adsorption behavior of the pollutants in MSW well [19]. So the adsorption rate is described by: 


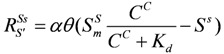
(30)

where 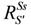 is the adsorption rate [MM^−^^1^]; 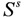 and 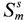 are the adsorption concentration and maximum adsorption concentration of IDS [MM^−^^1^];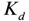 is the equilibrium sorption constant [ML^−^^3^]; 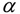 is the first order adsorption/desorption rate constant [T^−1^].

#### 2.4.6. Governing Equations

Based on the mass conservation principle and considering landfill settlement and hydrolysis of macromolecular organics, the governing equation for IDS can be described by:


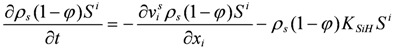
(31)

The SDS governing equation considering landfill settlement, hydrolysis of IDS and dissolution and biodegradation can be given by:


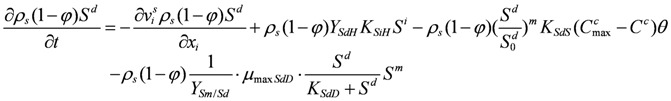
(32)

The MS governing equation considering landfill settlement, growth and decay of MS is described as:


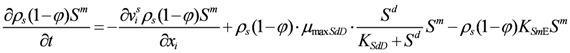
(33)

The AS governing equation is:


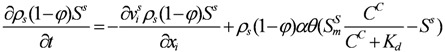
(34)

The SDA transport model considering convection and hydrodynamic dispersion, dissolution of SDS, aerobic and anaerobic degradation and adsorption/desorption is given by:


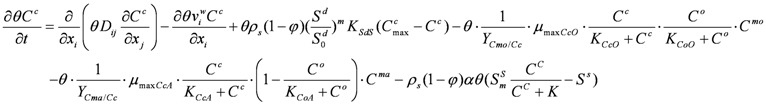
(35)

The equations for AM and ANM by considering convection and hydrodynamic dispersion, growth and decay are given by:


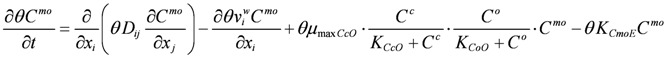
(36)


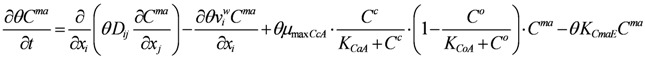
(37)

The governing equation for DO is:


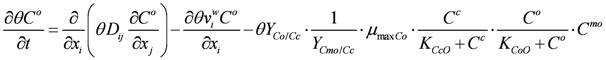
(38)

#### 2.4.7. Numerical Solution Method

The Merchant model was obtained by Lagrangian description. Hydraulic model and pollutant release and transport model were obtained by an Eulerian description. Total settlement in untreated landfilled MSW has been estimated to range between 25% and 50% of initial fill height [20]. So the upper boundaries of fluid and pollutant transport regions are obviously moving, and the small deformation assumption isn’t suitable. The coupling of these two types of model may lead to the moving boundaries of Eulerian describing models, so the Arbitrary Lagrangian-Eulerian (ALE) method was used to the model solution for solving the moving boundary problem. Due to space limitations, the solution process can be seen in the authors’ another work [15].

## 3. Results and Discussion

**Figure 2:**
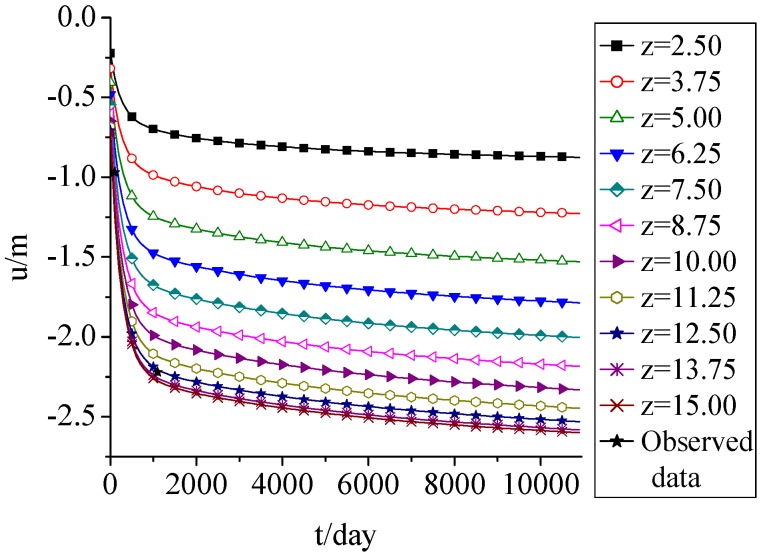
Displacement change with time.

An ideal landfill should have an effective seepage control system. After closure, it is in a relative independent state and can’t be affected by the external hydraulic environment. In this study, the change law of the main physical and chemical variables and its effect on the pollutant transport was analyzed in an ideal landfill. The simulated landfill had a rectangular vertical section of 15 m in height and 20 m in width. All boundaries were impervious. The upper boundary can move freely, and the others are all fixed. The parameters are given in Table 1. Physical and mechanical parameters were determined by testing, and biological parameters were obtained by parameter inversion. The results are shown in Section 3.1, Section 3.2, Section 3.3, Section 3.4, Section 3.5, Section 3.6, Section 3.7, Section 3.8, Section 3.9 and Section 3.10. In Figure 1 and Figure 2, z is the space coordinate of a certain particle at the initial time, that is, the corresponding space coordinate z at initial time was used to represent a certain particle. In the other figures, z is the space coordinates of a certain particle when the MSW was filled for 30 years, that is, the corresponding space coordinate z at 30 years after MSW was filled was used to represent a certain particle. 

**Table 1:** Model parameters.

Parameter	Values	Parameters	Values	Parameters	Values
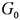	1,320 kPa	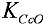	5.0 kg·m^−3^	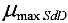	0.02 d^−1^
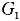	86.2 kPa	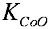	0.01 kg·m^−3^	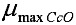	0.0005 d^−1^
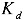	2.0×10^5^ d^−1^	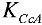	1.2 kg·m^−3^	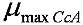	0.001 d^−1^
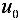	0 m	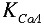	0.03 kg·m^−3^	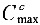	35,000 kg·m^−3^
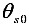	0.52	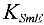	0.0001 d^−1^	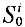	0 kg·kg^−1^
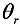	0.21	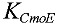	0.01 d^−1^	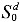	0 kg·kg^−1^
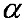	1.74 m^−1^	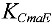	0.0002 d^−1^	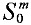	0 kg·kg^−1^
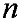	1.38	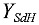	0.5	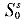	0 kg·kg^−1^
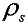	864 kg·m^−3^	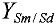	0.05	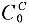	0 kg·m^−3^
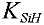	0.0006 d^−1^	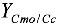	9×10^−5^	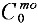	1.2×10^−4^ kg·m^−3^
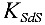	5×10^−9^ kg·kg^−1^	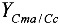	6×10^−5^	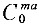	2.5×10^−6^ kg·m^−3^
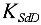	5.0 kg·kg^−1^	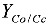	100	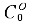	8×10^−3^ kg·m^−3^
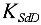	5.0 kg·kg^−1^	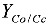			

### 3.1. Displacement

Figure 2 shows that the settlement occurred in almost 2 years. It’s about 85% of total settlement. The total settlement was about 2.6 m, which was about 17.3% of initial fill height. The simulation results fitted well with the observed data from a similar landfill cell in Wuhan Jinkou landfill in China which was observed from 2001 to 2010, and it accorded with the reported settlement law [21,22].

### 3.2. Porosity

**Figure 3:**
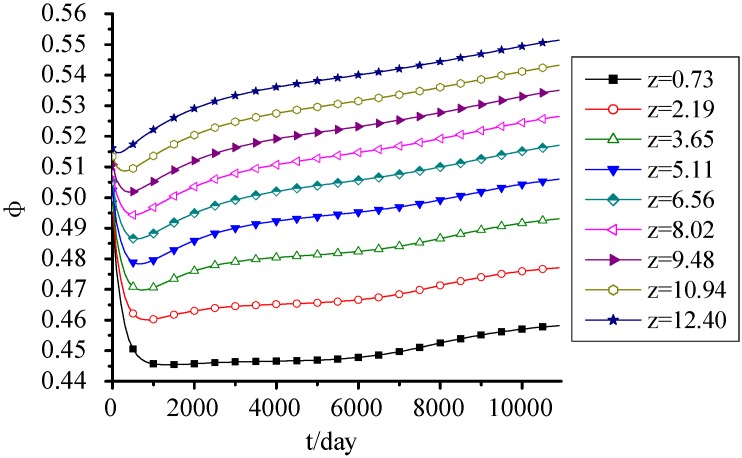
Porosity change with time.

Figure 3 shows that porosity decreased at first and then increased with time due to the landfill settlement and organic biodegradation. The 85% settlement that occurred in 2 years led to the MSW compression and the decrease of porosity. After 2 years, the effect of biodegradation on porosity was more and more obvious. The organic biodegradation led to the reduction of solid mass, and the porosity presented an increasing trend. When the MSW was filled for 30 years, the porosities at top and bottom increased to 0.55 and 0.458 again, respectively. Meanwhile, the decrease of the MSW porosity at bottom due to the landfill settlement was more obvious; however the increase of the one at top due to organic biodegradation was more obvious. In addition, the porosity presented an increasing trend with the fill height increasing. 

### 3.3. Saturated Hydraulic Conductivity

**Figure 4:**
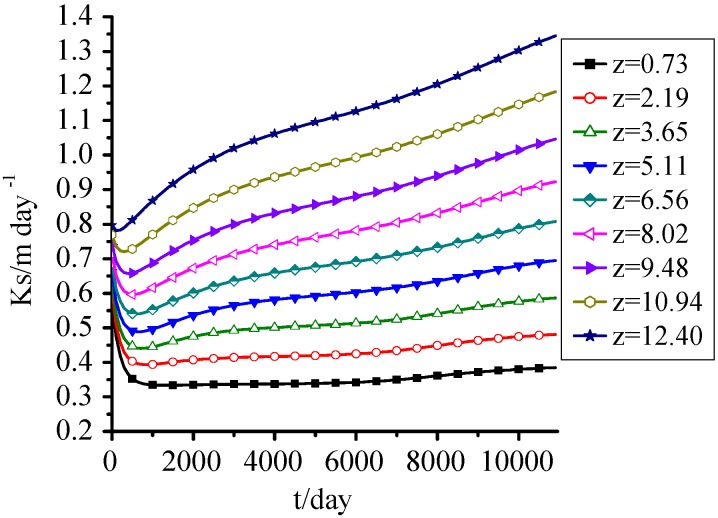
Ks change with time.

Figure 4 shows that the change of saturated hydraulic conductivity was similar to that of porosity, and the effect of displacement was significant. It decreased at first and then increased taking two years as a turning point. Increase was the main trend of Ks of the upper MSW, and the lower ones had a decreasing trend. The Ks at z = 0.73 m decreased from 0.8 m·day^−1^, which was the initial value, to 0.32 m·day^−1^, and then tended to be stable; and the one at z = 12.4 m showed a small decrease at first, and then increased until the maximum value.

### 3.4. Pressure Head

**Figure 5:**
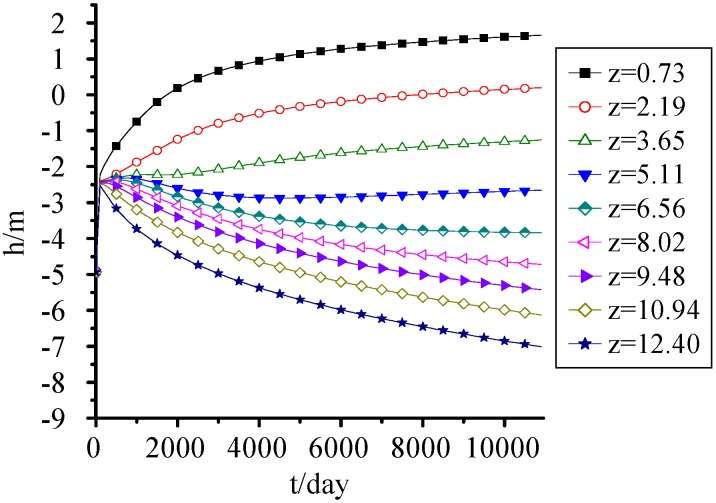
Pressure head change with time.

It's seen from Figure 5 the effect of settlement on pressure head wasn’t significant, although the water transport was closely related to landfill settlement, porosity and saturated hydraulic conductivity. The water mainly moved from top to bottom under gravity action and the upper pressure head decreased with time and the lower one increased by taking 5 m–6 m as separatrix. 

The pressure head at the bottom reached 0.6 m when MSW was filled for 5 years, and increased gradually with time. When MSW was filled for 30 years, the MSW below 2.5 m was saturated, which was equivalent to 16.7% of the landfill height. Thus, although without the effect of groundwater and surface water invasion, the landfill leachate generated by MSW itself was large, and can’t be neglected when designing the seepage control system and leachate treatment system.

### 3.5. IDS

**Figure 6:**
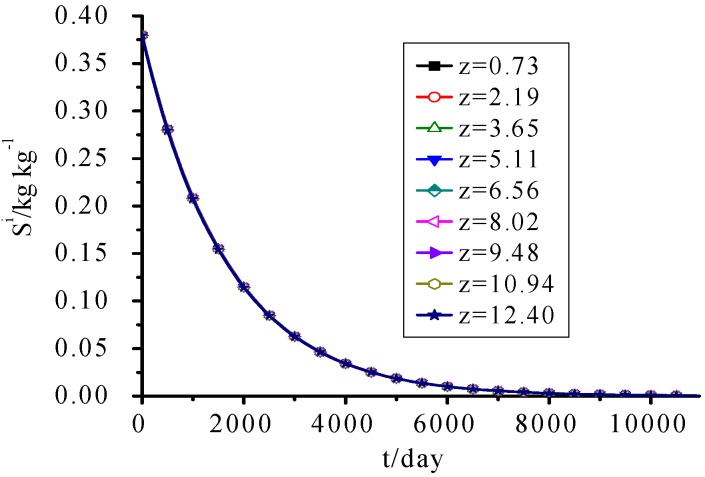
IDS Concentration change with time.

Figure 6 shows that because it’s assumed that the hydrolysis rate of IDS was only related to its own concentration, and the whole landfill cell has the same initial IDS concentration values, the calculated IDS concentration was only the decaying exponential function of time and independent of fill height and other parameters.

### 3.6. SDS

**Figure 7:**
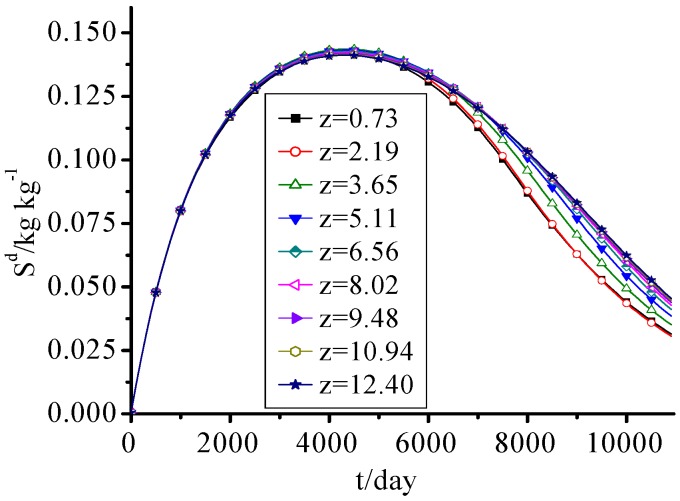
SDS concentration change with time.

SDS concentration in this landfill increased at first and then decreased with time as seen in Figure 7. This change process was closely related to the dissolution and biodegradation of SDS and the hydrolysis of IDS. Because of the fast hydrolysis of IDS in the early period of the landfill, the SDS concentration increased rapidly. Over time, IDS concentration gradually decreased, and SDS continuously dissolved from solid phase and was biodegraded, so the SDS concentration decreased year by year. The inflexion point was about the eleventh year.

The difference of SDS concentrations between every height was small and is more obvious before the MSW was filled for 15 years. Because the pore water of lower waste was tending to be saturated gradually, and the dissolution of soluble organic pollutants was accelerated, there was a little difference between upper waste and lower one after the MSW was filled for 15 years. 

### 3.7. MS

**Figure 8:**
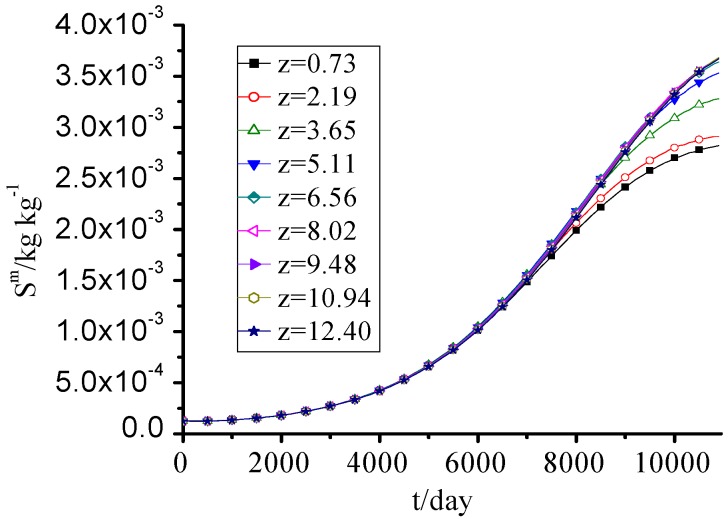
MS concentration change with time.

The MS concentration increased year by year, and the difference between every height was small as seen in Figure 8. 

### 3.8. SDA

**Figure 9:**
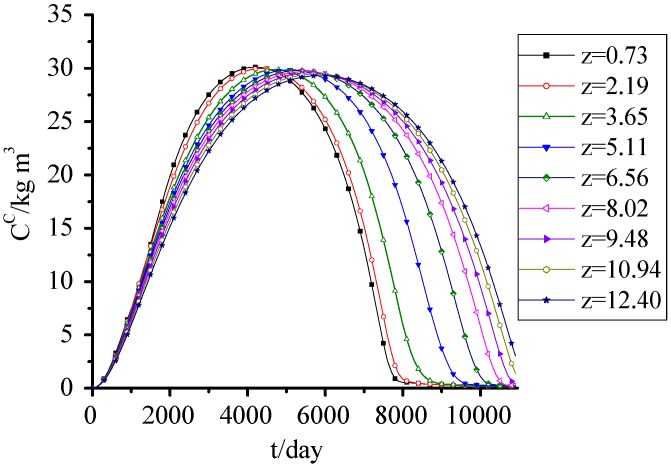
SDA concentration change with time.

Figure 9 shows the simulation results of SDA fitted well with the observed data from the Wuhan Jinkou landfill in China, which verified the accuracy of the model and parameters. These data were observed from 2001 to 2010. The suspended particles in water samples were filtered out and the SDA content was represented by chemical oxygen demand (COD) of the sample. SDA concentration increased at first and then decreased. At the early period, because of the higher SDS concentration and its dissolution to aqueous phase, the SDA concentration increased rapidly. Meanwhile, the water content of the lower waste was relatively higher, which accelerated the dissolution of SDS, so the SDA concentration in lower waste was higher than the upper one. The rapid dissolution of SDS in the early period also led to the higher SDA concentration in upper waste than the lower one in the later period of landfill.

### 3.9. ANM

**Figure 10:**
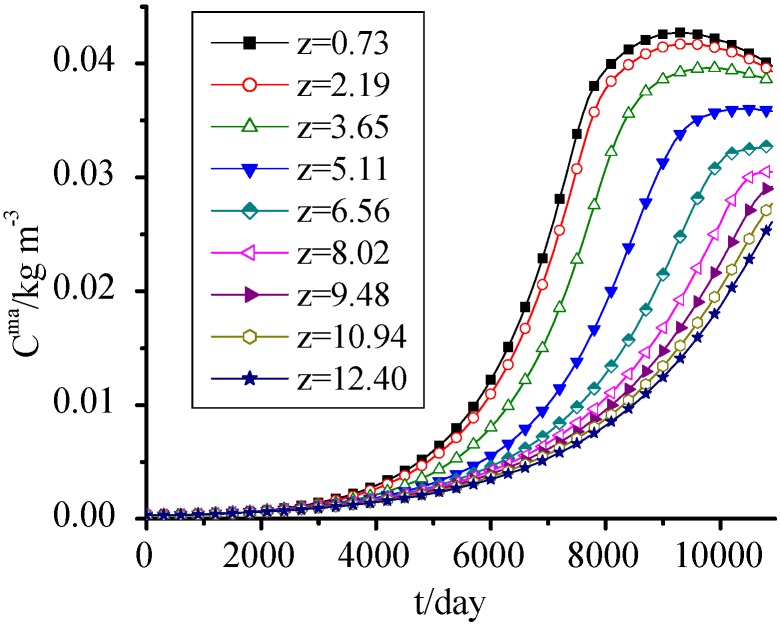
ANM concentration change with time.

It is seen from Figure 10 that the ANM concentration change was similar to that of MS in Figure 8, but it had a significant difference along height. The ANM concentration at z = 0.73 m was almost three times of the one at z = 12.40 m at utmost. This is mainly because the higher organic concentration in the lower waste provided sufficient nutrients for microorganisms and promoted their growth, while at later periods the SDA concentration in lower waste became lower, the ANM concentration presented a decreasing trend combining with its own decay.

### 3.10. AM and DO

**Figure 11:**
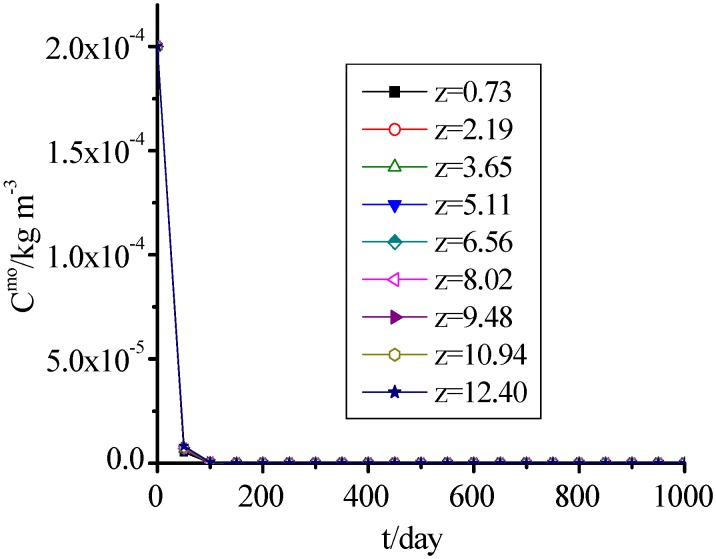
AM concentration change with time.

**Figure 12:**
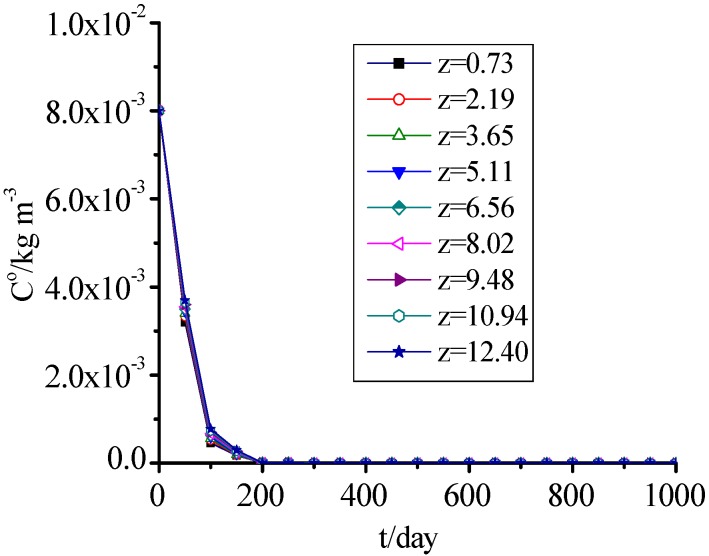
DO concentration change with time.

Figure 11 and Figure 12 show that the change of DO was consistent with AM, which concentrations presented a rapid decreasing trend with time, and reached 0 when MSW filled for 200 days. The values for the 1,001th day to 10,000th day were omitted in Figure 11 and Figure 12 because they were the same as on the 1,000th day.

## 4. Conclusions

A coupling kinetic model of landfill leachate pollutant release and transport in the process of landfill settlement was developed, which contained three sub models-landfill settlement model, hydraulic model and pollutant release and transport model. Landfill settlement, convection and hydrodynamic dispersion of leachate, hydrolysis, dissolution, adsorption/desorption, biodegradation of pollutant and other behaviors were considered. The release and transport of` pollutants and microorganisme in a landfill was simulated by considering no hydraulic action. The total settlement in this landfill cell was about 2.6 m, which was about 17.3% of initial height, and 85% almost occurred within 2 years. The simulation results fitted well with the observed data, and accorded with the reported settlement law. The changes of porosity and saturated hydraulic conductivity were closely related to settlement and biodegradation. They all presented a decreasing trend at first, and then increased with time. The leachate generated by MSW itself can saturated 16.7% of the landfill, so when designing the seepage control system and landfill leachate treatment system, this must be fully considered. The soluble and degradable organic pollutants in solid phase and aqueous phase presented an increasing trend at first and then decreased with time, respectively, due to the release of pollutants from the solid phase. The peak value of the latter could reach 30 kg·m^3^. The microorganisms in the solid phase and aqueous phase presented an increasing trend with time. 
